# Spectroscopic Study of Porphyrin-Caffeine Interactions

**DOI:** 10.1007/s10895-012-1090-9

**Published:** 2012-07-05

**Authors:** Magdalena Makarska-Bialokoz

**Affiliations:** Department of Inorganic Chemistry, Maria Curie-Sklodowska University, M. C. Sklodowska Sq. 2, 20-031 Lublin, Poland

**Keywords:** Caffeine, Porphyrin, Chlorophyll, Fluorescence quenching, Non-linear Stern-Volmer plots

## Abstract

The association between water-soluble porphyrins: 4,4′,4″,4‴-(21 *H*,23 *H*-porphine-5,10,15,20-tetrayl)tetrakis-(benzoic acid) (H_2_TCPP), 5,10,15,20-tetrakis(4-sulfonatophenyl)-21 *H*,23 *H*-porphine (H_2_TPPS_4_), 5,10,15,20-tetrakis[4-(trimethylammonio)phenyl]-21 *H*,23 *H*-porphine tetra-*p*-tosylate (H_2_TTMePP), 5,10,15,20-tetrakis(1-methyl-4-pyridyl)-21 *H*,23 *H*-porphine tetra-*p*-tosylate (H_2_TMePyP), the Cu(II) complexes of H_2_TTMePP and H_2_TMePyP, as well as chlorophyll a with caffeine (1,3,7-trimethylxanthine) has been studied analysing their absorption and emission spectra in aqueous (or acetone in case of chlorophyll a) solution. During the titration by caffeine the porphyrins absorption spectra undergo the evolution – the bathochromic effect can be observed as well as the hypochromicity of the Soret maximum. The association constants were calculated using curve-fitting procedure (K_AC_ of the order of magnitude of 10^3^ mol^-1^). Whereas the emission spectra point at the presence of the fluorescence quenching effect testifying for the partial inactivation of the porphyrin molecule. The fluorescence quenching constants were calculated from Stern-Volmer plots. The results obtained show that caffeine can interact with water-soluble porphyrins and through formation of stacking complexes is able to quench their ability to emission.

## Introduction

Caffeine (1,3,7-trimethylxanthine) is one of the most popular stimulants and an ingredient of many anaesthetic, anti-fever or dietary medicines. This compound is present as well in various plants where it acts as a natural pesticide, paralysing and killing insects that try to feed on the plant. Naturally occurring xanthines, like caffeine, are often included in the binding interceptor group [1]. It has been previously reported that methylxanthines are able to protect cells against the cytostatic and cytotoxic effects of some aromatic compounds by reduction of their mutagenic activity [2, 3]. Caffeine and other methylxanthines can form stacking complexes with several aromatic compounds, like anticancer drugs [2, 4], fluorescence dyes [5–9], mutagens [10, 11], neurotoxins [12, 13] and others [14, 15]. The authors of papers cited above assumed that hetero-association of methylxanthines with these compounds may diminish their biological activity. To reveal a possible mechanism that demonstrates the protective abilities of caffeine by stacking up and blocking the detrimental activity of aromatic mutagens and carcinogens UV–VIS spectroscopy and other techniques have been used [3].

In all products containing caffeine this compound occurs in the form of solution or water mixture. The gigantic consumption of caffeine means simultaneously the similar order of caffeine sewage production. The environment contamination caused by caffeine and its metabolites influences also the condition of water plant and animal organisms. Caffeine exposure induces as well early senescence in land plants and retards seedling growth [16]. The studies described in literature testify for the inhibiting influence of caffeine on the photosynthesis process of organisms leading to the decreasing of chlorophyll activity [16, 17]. It was found that the changes in chlorophyll fluorescence can be a cause of photosynthesis disturbance [18]. Therefore chlorophyll can be recognized not only as an indicator of the environment degradation due to contamination with heavy metals [19], but also as a sensitive biomarker of plant stress, taking into consideration that any kind of plant stress can affect plant growth as well as the process of photosynthesis.

Although caffeine is usually well-metabolized by human organism, its presence in surface water is considerable [20], particularly in the vicinity of inhabited areas, where this compound is delivered to the water environment in a continuous manner. It was found that the caffeine content is connected as well with the presence of human sewage in surface water. Sauve and co-workers proved that caffeine concentrations are relatively well correlated to cefal coliforms and could be potentially used as a chemical indicator of the level of contamination by sanitary sources and thereby could play a role of an anthropogenic marker [20, 21]. Therefore a precise detection of caffeine included in environmental samples can be significant for instance in monitoring of places with crude wastes thrown away out-of-control or drugs of abuse excreted unmetabolized or as metabolites to the sewage system [22].

**Fig. 1:**
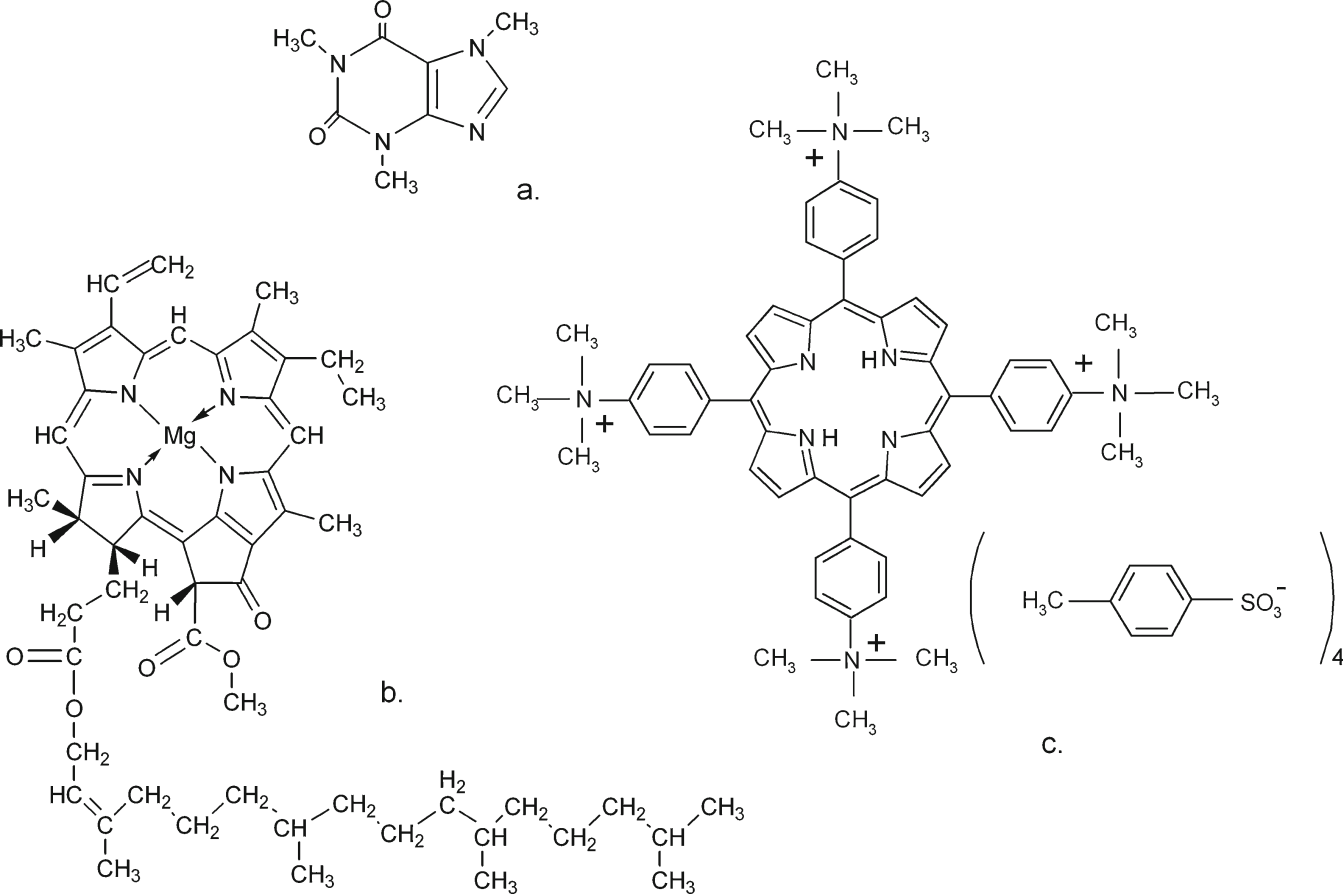
The molecular structures of (**a**) caffeine(1,3,7-trimethylxanthine), (**b**) chlorophyll a and (**c**) H_2_TTMePP (5,10,15,20-tetrakis[4-(trimethylammonio)phenyl]-21 *H*,23 *H*-porphine)

Studies described in this paper concern the spectroscopic analysis of interactions between biologically important macromolecules. The primary objective of presented research was to specify the mechanism of interactions of the chosen compounds from the class of water-soluble porphyrins with caffeine and verify as well their usefulness as chemical indicators of caffeine. The water-soluble porphyrins are the compounds with the specific spectroscopic and redox properties, as well as the ability to electron transfer, very sensitive to the subtle changes of pH, porphyrins and ligands concentration or form of complexing with metal ions proceeding in a reaction environment, what can be utilized among other things in their interactions with DNA [23–27], nucleic bases [28, M. Makarska-Bialokoz - unpublished results] and, what is equally important, in biomimetic catalysis [29–31] as well as in monitoring of the porphyrins interactions with different kinds of toxic substances [32, 33]. The secondary objective was to compare the behaviour of chlorophyll a (the porphyrin compound which is not soluble in water), during interaction with caffeine, to the results obtained for water-soluble porphyrins. To determine the caffeine - porphyrins relations the absorption and emission spectra evolution was observed during the titration by caffeine a series of water-soluble synthetic porphyrins: 5,10,15,20-tetrakis[4-(trimethylammonio)phenyl]porphine (H_2_TTMePP), 5,10,15,20-tetrakis(1-methyl-4-pyridyl)porphine (H_2_TMePyP), their complexes with Cu(II) (CuTTMePP and CuTMePyP), 4,4′,4″’,4‴-(21 *H*,23 *H*-porphine-5,10,15,20-tetrayl)tetrakis-(benzoic acid) (H_2_TCPP), 5,10,15,20-tetrakis(4-sulfonatophenyl)porphine) (H_2_TPPS_4_) and chlorophyll a (commercial reagent) (Fig. 1).

This paper presents, to the best of my knowledge for the first time, complex spectroscopic interaction analysis of water-soluble porphyrins and their copper (II) complexes with water solution of caffeine [M. Makarska-Bialokoz – personal communications: 2^nd^ International Conference on Multifunctional, Hybrid and Nanomaterials, HYMA, Strasbourg, France (06–10.03.2011)]. The association constants as well as the fluorescence quenching constants calculated for the examined systems could be recognized as well as the novelty.

## Experimental

### Reagents

Caffeine (1,3,7-trimethylxanthine) and the porphyrins: 4,4′,4″,4‴-(21 *H*,23 *H*-porphine-5,10,15,20-tetrayl)tetrakis-(benzoic acid) (H_2_TCPP, lg ε = 5.47, 415 nm); 5,10,15,20-tetrakis(4-sulfonatophenyl)-21 *H*,23 *H*-porphine (H_2_TPPS_4_, lg ε = 5.25, 413 nm); 5,10,15,20-tetrakis[4-(trimethylammonio)phenyl]-21 *H*,23 *H*-porphine tetra-*p*-tosylate (H_2_TTMePP, lg ε = 5.59, 412 nm) and 5,10,15,20-tetrakis(1-methyl-4-pyridyl)-21 *H*,23 *H*-porphine tetra-*p*-tosylate (H_2_TMePyP, lg ε = 5.32, 421 nm) were purchased in ALDRICH and used without any additional purification, whereas copper (II) complexes of H_2_TTMePP and H_2_TMePyP (CuTTMePP, lg ε = 5.49, 412 nm and CuTMePyP, lg ε = 5.34, 424 nm) were synthesized by the modification of the method described earlier in literature [34–36]. Chlorophyll a (lg ε = 5.13–5.23 for 428 nm and 5.05–5.14 for 660 nm) was also purchased in ALDRICH, while acetone in POCh S.A. Polskie Odczynniki Chemiczne.

### Measurements

The titration experiments were carried out using a 10^−3^ mol dm^−3^ stock solution of ligand (caffeine). The porphyrin solutions were freshly prepared in water (chlorophyll a solution in acetone) at the concentration range about 10^−7^ mol dm^−3^ to prepare the starting solution with the porphyrin absorbance value equals approximately 0.1. The initial volume of the porphyrin solutions used was 2 cm^3^. The volumes of the stock caffeine solution added at each step during titration of a porphyrin were as follows: 0, 0.005, 0.02, 0.03, 0.05, 0.1, 0.1, 0.2, 0.2, 0.2, 0.2 and 0.3 cm^3^ (final volume of stock caffeine solution was 1.405 cm^3^; final volume of solution in a cell was 3.405 cm^3^). The porphyrin concentration in case of H_2_TTMePP and chlorophyll a was changing in the range 3.27 – 1.92 10^-7^ mol dm^−3^ and 7.34 – 4.31 10^-7^ mol dm^−3^, respectively. The final concentration of caffeine in the mixture was 4.13 10^−4^ mol dm^−3^.

Absorption spectra were recorded on JASCO V-660 spectrophotometer, using 1 cm Hellma quartz cells to obtain spectra between 350 and 700 nm at the temperature of 21 °C. Emission spectra were recorded on JASCO FP-6300 spectrofluorometer. The database program Sigma Plot (version 9.0) (Jandel Corp.) was used in the manipulation and plotting of the data.

### Calculation of the Association Constants for Porphyrin - Caffeine Systems

To calculate the association (binding) constants the absorbance values in the Soret maximum were employed, because of higher comparing to Q band values of molar absorbance index, which permit to carry out the measurements at porphyrin concentration of the order of 10^−7^, what minimizes the dimerization process. The calculations were done using the Beck equation [37], which could be applied to obtained binding constants only on condition that the concentration of titrant is at least 100 times higher than the concentration of the compound examined.

For the determination of association constants of porphyrin – ligand (caffeine) complexes, according to reaction:

1 $$ P + L \leftrightarrow PL\overset{L}{\longleftrightarrow} P{{(L)}_{2}} + \ldots \overset{L}{\longleftrightarrow} P{{(L)}_{n}} $$

the equilibrium constant K_n_ can be written:

2 $$ {K_n} = \frac{{\left[ {P{{(L)}_n}} \right]}}{{\left[ {P{{(L)}_{{n - 1}}}} \right]\left[ L \right]}} $$

To calculate the final results the equation based on Bjerrum function modified by Beck [37] was applied:

3 $$ A = \frac{{{\varepsilon_0} + {\varepsilon_1}{K_1}\left[ L \right] + {\varepsilon_2}{K_1}{K_2}{{\left[ L \right]}^2} + ... + {\varepsilon_n}{K_1}{K_2}...{K_n}{{\left[ L \right]}^n}}}{{1 + {K_1}\left[ L \right] + {K_1}{K_2}{{\left[ L \right]}^2} + ... + {K_1}{K_2}...{K_n}{{\left[ L \right]}^n}}}\left[ P \right] $$

where *A* is the absorbance; *ε*_*0*_, the molar absorbance index for starting porphyrin; *ε*_*1*_ and *K*_*1*_, *ε*_*2*_ and *K*_*2*_, …, *etc.* are molar absorbance indexes and gradual binding constants for complexes with the stoichiometry 1:1, 1:2, …, *etc.*, respectively; *[L]* and *[P]* stand for the analytical concentration of ligand (caffeine) and porphyrin.

Taking into consideration the 1:1 model of complex formation, the values of K_1_ for all the porphyrins examined were determined by fitting the experimental data to Eq. (4), using the non-linear fitting procedure based on Marquardt–Levenberg algorithm (program Sigma Plot).

4 $$ A = \frac{{{\varepsilon_0} + {\varepsilon_1}{K_1}\left[ L \right]}}{{1 + {K_1}\left[ L \right]}}\left[ P \right] $$

The fitting procedure realized for 1:2 model did not make any physical sense.

### Calculation of the Fluorescence Quenching Constants for Porphyrin - Caffeine Systems

Quenching of fluorescence is usually described by the classic Stern-Volmer equation:

5 $$ \frac{{{{F}_{{\text{0}}}}}}{F} = 1 + {{K}_{{{\text{SV}}}}}\left[ Q \right] $$

where *F*_*0*_ and *F* are the fluorescence intensities in the absence and presence of quencher, respectively; *[Q]* is the concentration of quencher, *K*_*SV*_ is the Stern-Volmer quenching constant.

According to the experimental data, suggesting the presence of stacking interactions between caffeine and porphyrin systems, it was decided to determine the fluorescence quenching constants, taking into consideration primarily the process of static quenching [38]. To calculate the fluorescence quenching constants for all the systems examined the equation was applied

6 $$ \frac{{{{F}_{{\text{0}}}}}}{F} = 1 + {{K}_{{\text{S}}}}\left[ Q \right] $$

where *K*_*S*_ denotes the static quenching constant.

To calculate *K*_*S*_ the experimental data for each quencher concentration were fitted to Eq. (6) using the non-linear fitting procedure based on Marquardt–Levenberg algorithm (program Sigma Plot).

When the static quenching predominates, as in all probability in case of presented studies, a number of binding sites can be calculated

7 $$ \lg \frac{{{{F}_{{\text{0}}}} - F}}{F} = \lg {{K}_{{{\text{AC}}}}} + n\lg \left[ Q \right] $$

where *K*_*AC*_ is binding (association) constant, *n* is a number of binding sites, *[Q]* is the final concentration of quencher (caffeine), *F*_*0*_ and *F* are the fluorescence intensities for the porphyrin system in the absence and presence of quencher, respectively [39].

## Results and Discussion

### Analysis of Porphyrin - Caffeine Systems: UV–VIS and Fluorescence Spectra

The water-soluble porphyrin solutions were titrated by water in dilution experiment. All the systems examined behaved similarly and fulfilled a condition of the linearity of Beer-Lambert law. To avoid the concentration fluorescence quenching effect on emission spectra of porphyrins the measurements were carried out using the initial concentration of these compounds with the porphyrin absorbance values equal approximately 0.1. The phenomenon of concentration fluorescence quenching is known in chemistry since a long time [40] and connected with the fluorophore excess in a solution and with its diversified possibility to aggregation [41, 42], as well as with the polarity of examined system. The concentration quenching is particularly common for large aromatic molecules such as porphyrins, due to their ability to formation of dimers or bigger aggregates [43, 44], what can lead to partial or complete fluorescence decay, as a result of energy dissipation as well as re-absorption of emitted light.

**Fig. 2:**
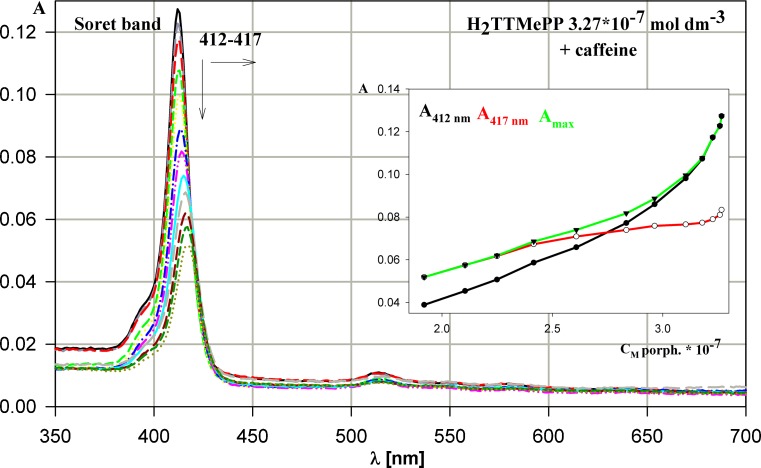
Evolution of H_2_TTMePP absorption spectrum during titration by caffeine. The dependence of absorbance versus porphyrin concentration for the process presented. The concentrations of the porphyrin and caffeine in solution changed in the range 3.27 – 1.92 (× 10^-7^ mol dm^-3^) and 0–4.13 × 10^-4^ mol dm^-3^, respectively

The evolution of absorption and emission spectra during the interactions between caffeine and a series of synthetic water-soluble porphyrins (H_2_TMePyP, CuTMePyP, H_2_TTMePP, CuTTMePP, H_2_TCPP, H_2_TPPS_4_) was recorded. In absorption spectra of H_2_TTMePP porphyrin the hypochromicity of the peak in Soret band and a shift towards the infrared (bathochromic effect, λ_max_ = 412–417 nm) can be observed (Fig. 2). In Q band the similar changes proceed, apparent particularly for IV band component (λ_max_ = 514–517 nm). For all the systems examined the dependence of absorbance *vs.* molar concentration of porphyrin shows the deviations from linearity confirming the existence of porphyrin – caffeine interactions.

**Fig. 3:**
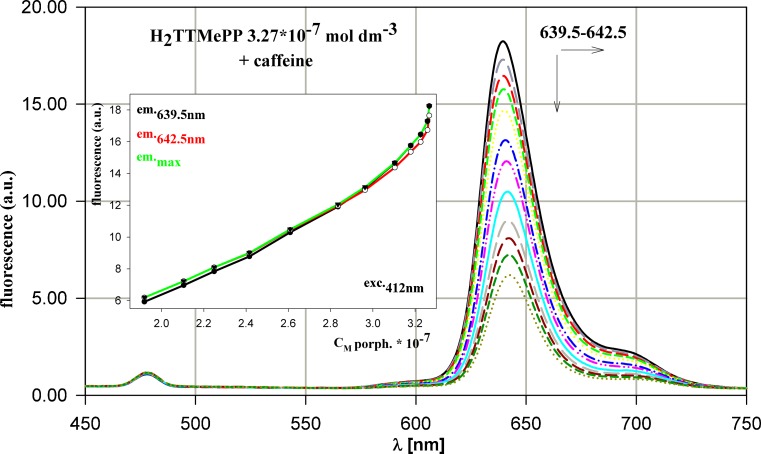
Evolution of H_2_TTMePP emission spectrum during titration by caffeine. The dependence of fluorescence intensity versus porphyrin concentration for the process presented. All the concentrations as in Fig. 2

In emission spectra, which undergo the bathochromic effect as absorption spectra, the decrease of peak maximum is observed, what testifies for the interactions with caffeine influencing the partial inactivation of the porphyrin and faster decay of its luminescence properties. In the same manner react H_2_TTMePP (Fig. 3), H_2_TCPP and H_2_TPPS_4_ porphyrins. While H_2_TMePyP is the only porphyrin, which in these experimental conditions is shifted towards the ultraviolet and shows the initial increase of the emission intensity.

Whereas the metalloporphyrins CuTTMePP and CuTMePyP present a different behaviour. The bathochromic shift of Soret band in absorption spectrum points at the interactions with caffeine, stronger in case of CuTTMePP. While the value of the emission intensity observed for these compounds is minimal, what testifies that the copper ions extinguish luminescence properties of the porphyrin complex. Such behaviour is typical for hypsochromic spectra of metalloporphyrins with Cu (II) ions [38, 45, 46]. The emission intensity of CuTTMePP complex is slightly higher, what is connected with a different structure of this complex. The big substituents of H_2_TTMePP porphyrin do not allow for entire hiding of Cu^2+^ ions in the porphyrin cave, forming the steric hindrance for the metal ions [47].

**Fig. 4:**
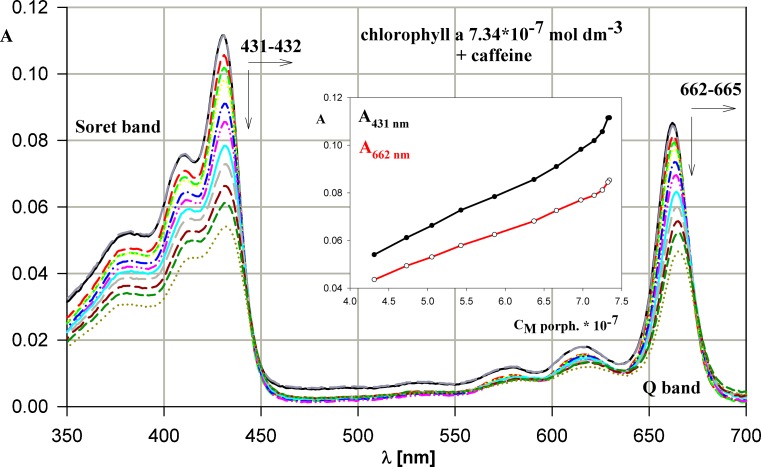
Evolution of chlorophyll a absorption spectrum during titration by caffeine. The dependence of absorbance versus chlorophyll a concentration for the process presented. The concentrations of chlorophyll a and caffeine in solution changed in the range 7.34 – 4.31 (× 10^-7^ mol dm^-3^) and 0–4.13 × 10^-4^ mol dm^-3^, respectively

Acetone solution of chlorophyll a was titrated both by acetone and water in order to separate the influence of these solvents on absorption and emission spectra of this compound. The slight deviations from Beer-Lambert law appearing in absorption spectrum of chlorophyll a (Fig. 4) can testify either for the minimal interactions with caffeine or for the time dependence occurring during this reaction or, what seems the most likely, for the different comparing to other porphyrins mechanism of interactions.

**Fig. 5:**
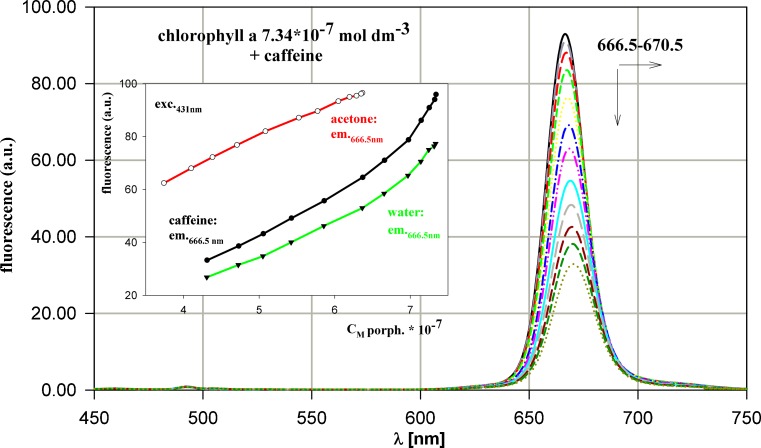
Evolution of chlorophyll a emission spectrum during titration by caffeine. The dependence of fluorescence intensity versus chlorophyll a concentration for titration by caffeine, water and acetone. All the concentrations as in Fig. 4

Both pure water and water solution of caffeine quench the fluorescence of chlorophyll a, as arises from the titration data (Fig. 5). The different mechanism of fluorescence quenching in case of this compound is probably the consequence of its structure, containing the phytol chain, which can hinder to some extent the binding of caffeine, as well as the form of a molecule, changing with the polarity of titrated solution. In acetone solution chlorophyll appears in monomeric form, due to interaction between central Mg in chlorophyll molecule, which acts as electron acceptor and carbonyl group in acetone molecule, which acts as electron donor [48]. Titration by water solution of caffeine increases the polarity of reaction environment, leading to formation of bigger aggregated molecules. Addition of water to acetone solution causes quenching of chlorophyll a fluorescence, what does not exclude the simultaneous interaction with caffeine. It was found that monomeric form of chlorophyll is less stable than dimeric (aggregated) form. In chlorophyll aggregates one chlorophyll may act as an electron donor and the other as electron acceptor via its central magnesium [48]. Therefore the obtained results point in this case at more than one mechanism of quenching and, possibly, more than one quenching centres [49].

### The Association Process in Porphyrin - Caffeine Systems

The values of association (binding) constants calculated for all the systems examined are presented in Table 1. The results point unequivocally at formation of associated systems between porphyrin compounds and caffeine. Hypochromic and bathochromic effects, occurring during the experiments, indicate that, upon the addition of caffeine to porphyrin solution, a new absorbing component appears in the mixture. Since both caffeine and porphyrin posses an aromatic structure, therefore they are capable of stacking complexes formation [1], founded on the interceptor molecule hypothesis postulated by Hartman and Shankel [50], which explains the existence of stacking interactions between the interceptor (flat ring system) and intercalator (polycyclic molecule).

**Table 1:** The binding (association) constants of associated molecules [mol^-1^] formed between porphyrins or their copper complexes and caffeine (error limits: ± 5 %)

porphyrin compound	absorbance maximum at Soret band [nm]	Soret band shift during titration with caffeine [nm]	binding constant [mol^-1^] × 10^3^	number of binding sites (n)
H_2_TTMePP	412.0	5.0	8.39	1.12
CuTTMePP	412.0	5.0	12.06	1.80
H_2_TMePyP	422.0	3.0	2.90^a^	1.31
CuTMePyP	425.0	1.0	10.61	1.65
H_2_TCPP	415.0	4.0	19.97	1.29
H_2_TPPS_4_	414.0	4.0	1.74^a^	1.00
chlorophyll a	430.0	1.0	11.15^a^	1.12

^a^ Error limits higher than 5 %

In discussed experiments higher values of association constants were obtained for the copper complexes comparing to free-base porphyrins, what have been already presented [51]. The values of *K*_*AC*_ for the compounds with 4-(trimethylammonio)phenyl groups (both free-base porphyrin and its copper complex) are higher comparing to the compounds with 1-methyl-4-pyridyl groups. It has been shown as well that the interactions of H_2_TTMePP with nucleic bases are much stronger than interactions of H_2_TMePyP what is most likely connected with the kind and the size of substituent porphyrin groups partaking in the process of stacking [M. Makarska-Bialokoz - unpublished results]. The charge of porphyrin substituent groups is also significant for the porphyrin – caffeine interactions. The cationic porphyrins seem to react stronger than the anionic ones (H_2_TPPS_4_). Nevertheless in case of H_2_TCPP porphyrin the highest value of *K*_*AC*_ is observed. Such result can be attributed to the different reaction environment - H_2_TCPP, which is hardly soluble in pure water, was dissolved in 0.01 mol dm^−3^ NaOH solution.

On the grounds of porphyrin – caffeine interactions analysis it was found that porphyrin compounds can react with caffeine molecule both by metal ions and hydrogen atoms presented in a porphyrin cave in case of complexes and free-base porphyrins, respectively, as well as by charged substituent groups. Caffeine interacts with porphyrins by a hydrogen bond as well as the interactions of an endocyclic nitrogen atom (N9) with a metal ion from a porphyrin cave. The confirmation of this postulate was presented by Fiammengo [51], describing the caffeine interactions with the system of conjugated with peptides zinc metalloporphyrin. The hydrophobic and hydrogen bonds interactions were as well the predominant intermolecular forces to stabilize the complex of caffeine with hemoglobin [39] and human serum albumin [52, 53] and the complex of amine with chlorophyll [54]. However, the complexity of chlorophyll molecule can lead to more composite interactions and formation of a system of different type, or even more than one system.

It should be considered that the structure of each porphyrin implies its ability to reach the excited state as well as the degree of the ring protonation process, what influences the manner of interaction with caffeine. Therefore the differences in the values of association constants are the consequence of the spatial structure of particular porphyrin compounds used in described experiments. Both the size and the charge of groups on the periphery of porphyrins as well as the type of porphyrin (free-base porphyrin or metalloporphyrin) and the axial-ligation of metalloporphyrins determine the position and a number of binding sites [47] (Table 1). Caffeine is known to dimerize or self-aggregate in aqueous solutions, but the association constants are very small (5–8 mol^−1^) and, therefore, do not affect the binding process [51]. At caffeine concentrations used in the experiments presented in this paper (10^−4^ mol dm^−3^) caffeine exists in the form of monomer [55].

### The Fluorescence Quenching Process in Porphyrin - Caffeine Systems

Formation of associated complexes, demonstrated by the calculated association constants *K*_*AC*_, confirms simultaneously the existence of static fluorescence quenching process in the porphyrin – caffeine system. Static quenching is often observed in case of stacking interactions between the numerous fluorophores and quenchers, particularly from the group of purine and pyrimidine nucleotides or related compounds [38]. It was already proved that caffeine could bind strongly with proteins (hemoglobin, albumin, lysozyme) [39, 53, 56, 57] and other systems at molar ratio 1:1 and a reaction is a single static quenching process. Formation of stacking complexes points unequivocally at static quenching proceeding in the discussed experiments. The values of fluorescence quenching constants calculated for all the systems examined are presented in Table 2.

**Table. 2:** The fluorescence quenching constants of associated molecules [mol^-1^] formed between porphyrins or their copper complexes and caffeine (error limits: ± 5 %)

porphyrin compound	emission intensity maximum [nm]	emission intensity shift during titration with caffeine [nm]	fluorescence quenching constant [mol^-1^] × 10^3^
H_2_TTMePP	639.5	3.0	4.36
CuTTMePP	639.0	---^b^	---^c^
H_2_TMePyP	650.0	2.0^a^	---^c^
CuTMePyP	635.5	---^b^	---^c^
H_2_TCPP	642.5	3.5	2.90
H_2_TPPS_4_	640.5	3.5	2.05
chlorophyll a	666.5	4.0	4.17^d^

^a^ hypsochromic shift

^b^ bathochromic shift (values of peak maximum on the level of background)

^c^ absence of quenching

^d^ quenching by water (predominantly)

The values of determined *K*_*S*_ confirm the statement that the extent of quenching depends on the structure and physicochemical properties of the fluorophore. The positive values of *K*_*S*_ constants were obtained only for H_2_TTMePP, chlorophyll a, H_2_TCPP and H_2_TPPS_4_. However, the quenching of chlorophyll a could be attributed both to water and caffeine. The obtained results indicate most likely the existence of at least two simultaneous processes, difficult to separate, proceeding in the system: (a) dilution of acetone solution of chlorophyll a by water, connected both with the aggregation process and quenching activity of water, as well as (b) quenching of chlorophyll a fluorescence intensity by caffeine, resulting from the formation of associated complexes. In the event of CuTTMePP and CuTMePyP complexes only the inconsiderable emission can be observed which could be attributed to the difference in their molecular geometry and lack of charge transfer state in molecules [58]. In case of H_2_TMePyP porphyrin initially an increase of fluorescence is observed and subsequently the slight decrease of emission, what is most likely the consequence of its molecular structure.

**Fig. 6:**
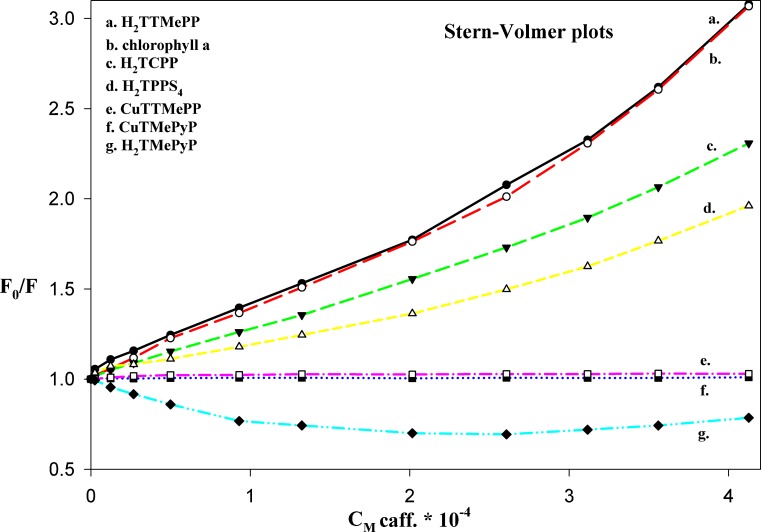
Stern-Volmer plots for all the porphyrin compounds examined during titration by caffeine

The Stern-Volmer plots would be linear within certain concentration if the quenching type is single static [39]. Then the values of *K*_*S*_ should correspond to the values of *K*_*AC*_ [38]. If the quenching type is combined (both static and dynamic), the Stern-Volmer plot is an upward curvature, what can be observed in case of presented results (Fig. 6). On the other hand the values of calculated fluorescence quenching constants are predominantly lower comparing to *K*_*AC*_ values and too large to be due to collisional (dynamic) quenching, what confirms simultaneously that caffeine must be bound to porphyrin. Therefore the observed quenching process can not be elucidated by the simple static or dynamic quenching process, or combination of them.

The emission data show that the fluorescence intensity of examined porphyrins decreases with the increasing concentration of caffeine, resulting in a bathochromic shift of the peak in case of majority of porphyrins and in a hypsochromic shift as well as a slight change of the peak shape in case of H_2_TMePyP porphyrin. It was found that some quenchers are known to bind to protein and induce conformational changes [38]. The change of a molecule shape causes not only the decrease of emission peak, but also its shift. Such behaviour of quenched compounds, observed in presented experiments, confirms as well the static quenching mechanism.

Non-linear Stern-Volmer plots with an inflexion point at the caffeine concentration equals approximately 2.0 – 2.5∙10^−4^ mol dm^−3^ are characteristic of all quenched by caffeine porphyrins. Similar Stern-Volmer plots, composed of two line segments, were presented by Wang [49]. Such type of plot can indicate that the interaction mechanism becomes more complex, what can point at the presence of specific binding interactions, connected with more than one form of fluorophore, with at least one form undergoing the quenching process, or more than one biding site in the neighbourhood of the fluorophore. To sum up, all the results show that in presented systems there are obviously characters of static quenching, connected with the formation of a ground-state complexes with caffeine, accompanied simultaneously by the additional specific binding interactions.

## Conclusions

1. Bathochromic and hypochromic shift of Soret band maximum in absorption spectra and calculation results discussed in this paper indicate the presence of direct stacking interactions between caffeine and all porphyrins examined. For all tested compounds increasing concentration of porphyrin - caffeine stacking aggregates is associated with the decline in the concentration of the free active form of porphyrin in the mixture. Calculated association constants values are in good agreement with *K*_*AC*_ values determined previously for several aromatic compound - caffeine systems [1, 4, 5, 8].
2. Fluorescence quenching in emission spectra points at the decrease of luminescence properties of water-soluble porphyrins examined and can be predominantly attributed to the process of static quenching. The order of calculated fluorescence quenching constants values is in good agreement with data presented previously in literature [38]. The most distinct decay of fluorescence intensity can be observed in case of H_2_TTMePP porphyrin.
3. The obtained data can be applied in determination of porphyrin interactions and their decay kinetics. The results could become as well a base for the elaboration of a new artificial caffeine sensor, potentially useful for monitoring of caffeine sewage in aqueous environment.
